# Adaptive control for a class of nonlinear complex dynamical systems with uncertain complex parameters and perturbations

**DOI:** 10.1371/journal.pone.0175730

**Published:** 2017-05-03

**Authors:** Jian Liu, Kexin Liu, Shutang Liu

**Affiliations:** 1 School of Mathematical Sciences, University of Ji’nan, Ji’nan, Shandong 250022, P. R. China; 2 Academy of Mathematics and Systems Science, Chinese Academy of Sciences, Beijing 100190, P. R. China; 3 School of Control Science and Engineering, Shandong University, Ji’nan, Shandong 250061, P. R. China; Lanzhou University of Technology, CHINA

## Abstract

In this paper, adaptive control is extended from real space to complex space, resulting in a new control scheme for a class of *n*-dimensional time-dependent strict-feedback complex-variable chaotic (hyperchaotic) systems (CVCSs) in the presence of uncertain complex parameters and perturbations, which has not been previously reported in the literature. In detail, we have developed a unified framework for designing the adaptive complex scalar controller to ensure this type of CVCSs asymptotically stable and for selecting complex update laws to estimate unknown complex parameters. In particular, combining Lyapunov functions dependent on complex-valued vectors and back-stepping technique, sufficient criteria on stabilization of CVCSs are derived in the sense of Wirtinger calculus in complex space. Finally, numerical simulation is presented to validate our theoretical results.

## Introduction

Chaos is a ubiquitous phenomenon in nonlinear system. Over the last few decades, the chaotic behavior has been discovered in numerous systems in atmosphere [1], chemistry [2], biology [3], laser [4], mechanics [5], electronic circuits [6], and so on. It is well known that chaos effect may be undesirable in practice, it is often necessary that chaos should be controlled so that the system trajectory exhibits a desired dynamics. Therefore, chaos control plays a very important role in many different contexts. After the pioneering work of Ott, Grebogi and Yorke (OGY) [7] in 1990, chaos control and synchronization have attracted increasing attention in academic research and practical applications. For example, Petrov et al. [8] stabilized periodic behavior embedded in chaotic attractor of the BZ reaction by proportional-feedback. Pyragas [9] controlled chaos via an unstable delayed feedback controller. Wang and Lin [10] developed an observer-based fuzzy neural sliding mode control scheme for interconnected unknown chaotic systems. Wang et al. [11] presented networked synchronization control of coupled dynamic networks with time-varying delay. Luo and Zeng [12] investigated adaptive control of unknown strict-feedback chaotic systems by introducing proper auxiliary variable.Particularly, backstepping has become one of the most popular design methods for nonlinear control because it can guarantee global stabilities, tracking and transient performance for a broad class of nonlinear systems. For instance, Lü and Zhang [13] proposed backstepping design for controlling Chen’s chaotic attractor based on parameters identification. Park [14] proposed master-slave synchronization of Genesio chaotic system via backstepping approach. Wu et al. [15] stabilized a class of nonlinear strict-feedback time-delay systems by an adaptive backstepping neural controller. However, these fruits are all in real space.

In complex space, Fowler et al. [16, 17] derived originally the Lorenz equations with complex variables and complex parameters to describe rotating fluids and ring laser in 1982. Twenty-five years later, Mahmoud et al. [18] introduced Chen and Lü complex-variable chaotic systems (CVCSs) with real parameters. Liu and Liu [19] presented the adaptive anti-synchronization of CVCSs with unknown real parameters. Wang et al. [20–26] realized module-phase synchronization, modified function projective lag synchronization, hybrid modified function projective synchronization, and complex generalized synchronization of CVCSs or neural networks.

**Fig 1:**
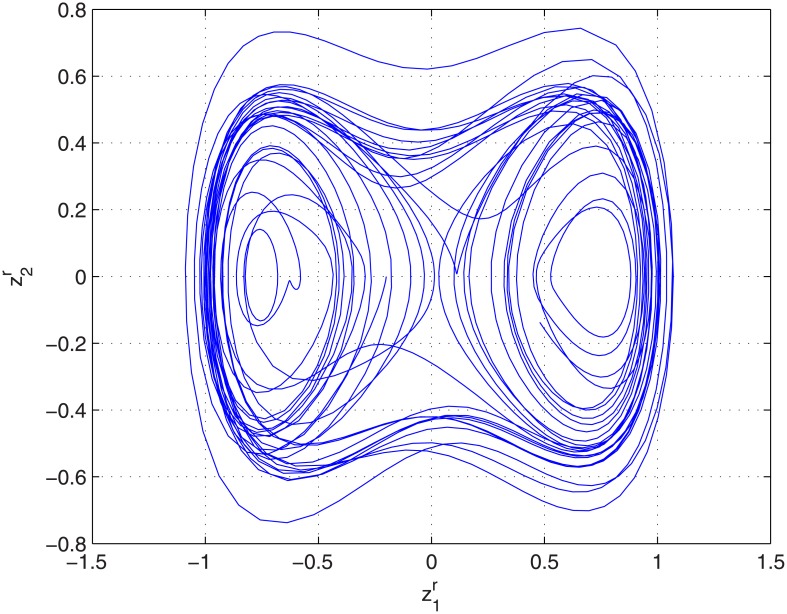
The phase portrait of the chaotic attractor for unperturbed Duffing CVCSs Eq (1) with complex parameters *α* = 0.13, *β* = *w* = 1, *γ*′ = 0.18(1 + *j*), and initial values *z*_1_ = −0.2 − 0.2*j*, *z*_2_ = 0.

As is known to all, complex-variable Duffing’s oscillator appear in many important fields of physics and engineering, for example, in nonlinear optics, deep-water wave theory, plasma physics and bimolecular dynamics. The complex-variable Duffing’s oscillator model [27] can be expressed in the form of strict-feedback CVCSs with complex parameter, which is given by

$$\begin{array}{c} \left\{\begin{array}{c} {\dot{z}}_{1}=z_{2},\\ {\dot{z}}_{2}=f\left(z_{1},z_{2},{\bar{z}}_{1},{\bar{z}}_{2},t\right), \end{array}\right. \end{array}$$ (1)

where $z_{1}=z_{1}^{r}+jz_{1}^{i},z_{2}=z_{2}^{r}+jz_{2}^{i}$ are complex-valued state variables, $f=z_{1}-\alpha z_{2}-\beta z_{1}^{2}{\bar{z}}_{1}+\gamma^{\prime }cos\left(\omega t\right)$, $\gamma^{\prime }=\sqrt{2}\gamma \exp\left(\frac{j\pi }{4}\right)$, *α*, *β*, *γ* and *ω* are positive parameters, and a dot is time derivative, chaotic motion of complex system (1) is shown as in Fig 1. In fact, a variety of physical systems could be written as the form of strict-feedback CVCSs, such as perturbed van der Pol CVCSs [28], Jerk CVCSs [29]. Up to now, there have been only a few papers on the stabilization for strict-feedback CVCSs. For example, the Duffing CVCSs (1) in [27] is stabilized by using the method in reference [9] in 2001. The chaos control of van der Pol CVCSs which occurs in vacuum tube circuits [28] is achieved by using a feedback control method in 2008, and that of jerk CVCSs [29] are investigated by adding a complex periodic forcing in 2012.

Surprising, it is found that the state variables in the mentioned studies are all supposed to be real-valued [8–15] or complex-valued with real parameters [18–26]. As is well known, complex nonlinear dynamic systems are more complicated than real systems, and can generate more abundant dynamical behaviors, which can be applied to secure communication for high transmission efficiency and anti-attack ability. In addition, the complex parameters in CVCSs follow from purely physical consideration, for example, complex parameters in [16, 17] arise due to the weak dispersive effects and are related to the detuning, we should consider the effect of the detuning in many practical applications. Very recently, Liu et al. [30–33] investigated several kinds of complex projective synchronization for a class of CVCSs with complex parameters. To the best of our knowledge, however, fewer works have been done to study the stabilization problems for strict-feedback CVCSs involving complex parameters, such as system (1).

On the other hand, in many practical engineering problems, it is hard to assume that all the exactly values of system parameter are known a priori, and sometimes there are also perturbations in system, and the system may be time-dependent. To deal with these unknown factors, adaptive control has been widely used as an effective method. Furthermore, most of the publications concern on complex chaos control and synchronization are only valid for some particular strict-feedback CVCSs, and their Lyapunov stabilization are not investigated [27–29]. However, as mentioned in [12], from the viewpoint of practical applications, it is expected that the control and synchronization scheme can be used for more CVCSs. As far as we know, there are no achievements about stabilization of time-dependent strict-feedback CVCSs with uncertain complex parameters and perturbations. Therefore, how to stabilize this kind of CVCSs via the adaptive control in complex space is an open problem.

Inspired by the aforementioned discussions, in this paper our major concerns are adaptive control for time-dependent strict-feedback CVCSs in the presence of uncertain complex parameters and perturbations by using backstepping approach. Compared with the previous works, the main contributions of the present paper are summarized as follows.

First, the systems under investigation are remarkably more general than those in the closely related literatures [12, 14, 15, 27–29]. In [12, 15], stabilization of strict-feedback real-variable chaotic system (RVCSs) was investigated. In [14], synchronization of Genesio RVCSs was achieved. It is well known that Genesio RVCSs is a special strict-feedback chaotic system, and real space is a subspace of complex one. Moreover, the authors [27–29] only investigated non-Lyapunov stabilization of some particular strict-feedback CVCSs, such as Duffing, van der Pol and Jerk CVCSs. However, Lyapunov stabilization problem has not been solved for more general strict-feedback CVCSs Eq (7) in complex space. In the present work, we address a unified framework for adaptive control of time-dependent strict-feedback CVCSs with uncertain complex parameters and perturbations.

Second, in contrast to the classical control and synchronization schemes proposed in the literature [18–29], we accomplish all the theoretical works in the sense of Wirtinger calculus in complex space [34–36]. From the technical perspective, as described in [30–33], the classical control and synchronization for CVCSs, which were all achieved by separating imaginary and real parts of complex variables, are still that of RVCSs. As is well known, there are essential differences between RVCSs and CVCSs. Most properties and conclusions of RVCSs cannot be simply extended to that of the CVCSs. What’s more, it is difficult or even impossible to separate imaginary and real parts of complex variables for some CVCSs. To avoid this limitation, we use Wirtinger calculus in this paper, extend adaptive control from real space to complex space, and accomplish all the theoretical works in complex space. Combining Lyapunov functions dependent on complex-valued vectors and back-stepping technique, stabilization of this type of CVCSs is achieved by complex scalar adaptive controller, unknown complex parameters are estimated by complex update laws.

The rest of this paper is organized as follows. In section 2, some preliminaries and relevant lemmas are briefly reviewed. In section 3, problem formulation and some assumptions are given. In Section 4, back-stepping method is employed and the adaptive complex scalar controller is designed and the complex update laws of unknown parameters are selected. A numerical example is presented in Section 5. Finally, Section 6 draws some conclusions.

## Preliminaries

**Notation** The notations used throughout the paper are standard. $C^{n}$ stands for *n* dimensional complex vector space. If $z\in C^{n}$ is a complex vector, then **z** = **z**^*r*^ + *j***z**^*i*^, $j=\sqrt{-1}$ is the imaginary unit, superscripts *r* and *i* stand for the real and imaginary parts of **z**, respectively; **z**^T^ and **z**^H^ are the transpose, conjugate transpose of **z**, respectively, and ‖**z**‖ implies the 2-norm of **z**. If *z* is a complex scalar, |*z*| indicates the modulus of *z* and $\bar{z}$ is the conjugate of *z*. $\hat{\Theta }$ is the estimation of complex parameter vector **Θ**.

### Wirtinger calculus

In this subsection, we first recall briefly the definition of Wirtinger calculus and some basic facts. As stated in [34–36], Wirtinger calculus also called the CR calculus, which provides a framework for differentiating nonanalytic functions. Importantly, it allows performing all the derivations in complex field, in a manner very similar to the real-valued case.

In classical complex-variable theory, as the differentiation of $\bar{z}$ by *z* is not defined, the function ϕ:C→C given by $\phi \left(z\right)=\bar{z}$ is not analytic; i.e. is not differentiable with respect to *z* in the Cauchy-Riemann sense. Thus the real-valued function f:C→R given by $f\left(z\right)=z\bar{z}={\left|z\right|}^{2}$ is not analytic in the Cauchy-Riemann sense either. To avoid this limitation, it is convenient to define a generalization or extension of the standard partial derivative to nonholomorphic functions of complex-valued variable *z* = *z*^*r*^ + *jz*^*i*^, that is differential with respect to *z*^*r*^ and *z*^*i*^. Generally speaking, nonholomorphic functions *F*(*z*) can be viewed as $F(z,\bar{z})$, where they are holomorphic in *z* for fixed $\bar{z}$ and holomorphic in $\bar{z}$ for fixed *z*. This underlies the development of Wirtinger calculus. Associated with these functions are two partial derivatives ${\partial F/\partial z=\partial F/\partial z|}_{\bar{z}=constant}$ and $\partial F/\partial \bar{z}=\partial F/\partial \bar{z}{|}_{z=constant}$ that are given by

$$\frac{\partial F}{\partial z}=\frac{1}{2}\left(\frac{\partial F}{\partial z^{r}}-j\frac{\partial F}{\partial z^{i}}\right),\;\;\;\frac{\partial F}{\partial \bar{z}}=\frac{1}{2}\left(\frac{\partial F}{\partial z^{r}}+j\frac{\partial F}{\partial z^{i}}\right).$$ (2)

Note that from Eq (2) that we immediately have the properties

$$\frac{\partial z}{\partial z}=\frac{\partial \bar{z}}{\partial \bar{z}}=1,\;\;\;\frac{\partial z}{\partial \bar{z}}=\frac{\partial \bar{z}}{\partial z}=0.$$ (3)

The differential form of a function with respect to complex variables *z* and $\bar{z}$ is

$$dF\left(z,\bar{z}\right)=\left(\frac{\partial F}{\partial z}\right)dz+\left(\frac{\partial F}{\partial \bar{z}}\right)d\bar{z},$$ (4)

Generalizing the above concept to complex domain vector space, the differential form of a function with respect to complex column vectors $z,\bar{z}\in C^{n}$ is

$$dF\left(z,\bar{z}\right)={\left(\frac{\partial F}{\partial z}\right)}^{T}dz+{\left(\frac{\partial F}{\partial \bar{z}}\right)}^{T}d\bar{z},$$ (5)

where the complex gradient and complex conjugate gradient operators for complex column vectors are defined respectively as

$$\nabla_{z}=\frac{\partial }{\partial z}≜{\left(\frac{\partial }{\partial z_{1}},\frac{\partial }{\partial z_{2}},\ldots ,\frac{\partial }{\partial z_{n}}\right)}^{T},$$

$$\nabla_{\bar{z}}=\frac{\partial }{\partial \bar{z}}≜{\left(\frac{\partial }{\partial {\bar{z}}_{1}},\frac{\partial }{\partial {\bar{z}}_{2}},\ldots ,\frac{\partial }{\partial {\bar{z}}_{n}}\right)}^{T}.$$

### Relevant lemmas

**Lemma 1.** [36]

If $F:C^{n}\to R$ be a real-valued function of a complex vector **z**, let $F\left(z\right)=F\left(z,\bar{z}\right)$, then

$$\frac{\partial F}{\partial \bar{z}}=\bar{\left(\frac{\partial F}{\partial z}\right)}.$$ (6)

**lemma 2.** (Barbălat’s lemma [37]) If the differentiable function *f*(*t*) has a finite limit, as *t* → ∞, and if $\dot{f}\left(t\right)$ is uniformly continuous (a sufficient condition for a differentiable function to be uniformly continuous is that its derivative is bounded), then $\dot{f}\left(t\right)\to 0$, as *t* → ∞.

## Problem formulation and assumptions

In this paper, we consider a class of *n*-dimensional time-dependent strict-feedback CVCSs as follows

$$\begin{array}{c} \left\{\begin{array}{c} {\dot{z}}_{1}=z_{2}+\Psi_{1}^{H}\left(z_{1},{\bar{z}}_{1}\right)\Theta ,\\ {\dot{z}}_{2}=z_{3}+\Psi_{2}^{H}\left(z_{1},z_{2},{\bar{z}}_{1},{\bar{z}}_{2}\right)\Theta ,\\ \;\;\;...,\\ {\dot{z}}_{n-1}=z_{n}+\Psi_{n-1}^{H}\left(z_{1},z_{2},...,z_{n-1},{\bar{z}}_{1},...,{\bar{z}}_{n-1}\right)\Theta ,\\ {\dot{z}}_{n}=\varphi \left(z,\bar{z},t\right)+\Psi_{n}^{H}\left(z,\bar{z}\right)\Theta +u\left(t\right), \end{array}\right. \end{array}$$ (7)

where $z={\left(z_{1},z_{2},...,z_{n}\right)}^{T}\in C^{n}$ is complex state vector, $\varphi :C^{n}\times C^{n}\times \left[0,+\infty \right)\to C$ is a nonlinear scalar complex-valued function, $\Psi_{i}:C^{i}\times C^{i}\times \left[0,+\infty \right)\to C^{s},\left(i=1,2,...,n\right)$ are known complex-valued function vectors, respectively. $\Theta ={\left(\theta_{1},\theta_{2},...,\theta_{s}\right)}^{T}\in C^{s}$ is an uncertain complex parameter vector, and u(t)∈C is the complex scalar control input. In the following, $\Psi_{i}\left(z_{1},z_{2},...,z_{i},{\bar{z}}_{1},{\bar{z}}_{2},...,\bar{z_{i}}\right)$ will be replaced by **Ψ**_*i*_ for convenience. The aim of this paper is to design proper control input u(t)∈C and complex update law of **Θ** to ensure the global stability of the CVCSs Eq (7).

**Remark 1.** It is well known that the strict-feedback RVCSs are described as

$$\begin{array}{c} \left\{\begin{array}{c} {\dot{x}}_{1}=x_{2}+{ϒ}_{1}^{T}\left(x_{1}\right)\Theta ,\\ {\dot{x}}_{2}=x_{3}+{ϒ}_{2}^{T}\left(x_{1},x_{2}\right)\Theta ,\\ \;\;\;...,\\ {\dot{x}}_{n-1}=x_{n}+{ϒ}_{n-1}^{T}\left(x_{1},x_{2},...,x_{n-1}\right)\Theta ,\\ {\dot{x}}_{n}=f\left(x\right)+{ϒ}_{n}^{T}\left(x\right)\Theta +u\left(t\right), \end{array}\right. \end{array}$$ (8)

where $x={\left(x_{1},x_{2},...,x_{n}\right)}^{T}\in R^{n}$ is real-valued state vector, *f*, **ϒ**_*i*_ (*i* = 1, 2, …, *n*) are real-valued smooth scalar function and function vectors respectively, and $\Theta ={\left(\theta_{1},\theta_{2},...,\theta_{s}\right)}^{T}\in R^{s}$ is an uncertain real-valued parameter vector, u(t)∈R is the real-valued scalar control input. In [12], Luo and Zeng investigated the control of chaotic system (8) in the absence of **ϒ**_*i*_ (*i* = 1, 2, …, *n* − 1), which is the special case of system (8). Obviously, system (7) is the complex-valued extension of system (8).

Throughout this paper, we will assume as follows.

**Assumption 1.** **Ψ**_*i*_ has *n* − 1 order partial derivatives with respect to complex vectors $\left(z_{1},z_{2},...,z_{i}\right),\left({\bar{z}}_{1},{\bar{z}}_{2},...,{\bar{z}}_{i}\right)\in C^{n}$. Moreover, **Ψ**_*i*_(0) = **0**, *i* = 1, 2, …, *n*.

**Assumption 2.** The unknown complex parameter vector **Θ** is norm-bounded, i.e., there exists *d* > 0, such that ‖**Θ**‖ < *d*.

## Main results

In the following, we consider the general case that **Ψ**_*i*_ ≠ **0** (*i* = 1, 2, …, *n*).

### Adaptive complex scalar controller design based on back-stepping

In this subsection, we employ the adaptive back-stepping control technique to design our complex scalar controller and complex update laws for *n*-dimensional CVCSs. The designing procedure is achieved by *n* steps.

**Step 1.** First, let us analyze the subsystem

$${\dot{z}}_{1}=z_{2}+\Psi_{1}^{H}\Theta .$$ (9)

Consider the Lyapunov function candidate defined on complex space in the form as

$$V_{1}=\frac{1}{2}\left[{\bar{z}}_{1}z_{1}+{\left(\Theta -\hat{\Theta }\right)}^{H}\left(\Theta -\hat{\Theta }\right)\right].\;\;\;$$ (10)

Let

$$\begin{array}{c} \left\{\begin{array}{ccc} \phi_{1} & = & -Mz_{1}-\Psi_{1}^{H}\hat{\Theta },\\ \Lambda_{1} & = & z_{1}\Psi_{1}, \end{array}\right. \end{array}$$ (11)

where *M* > 0, and according to Lemma 1 and the chain rule, the time derivative of *V*_1_ along the subsystem Eq (9) is given by

$$\begin{array}{lll} {\dot{V}}_{1} & = & \frac{1}{2}\left[{\bar{z}}_{1}\left(z_{2}-\phi_{1}+\phi_{1}+\Psi_{1}^{\text{H}}\Theta \right)+z_{1}{\dot{\bar{z}}}_{1}\right]-\frac{1}{2}\left[{\left(\Theta -\hat{\Theta }\right)}^{\text{H}}\dot{\hat{\Theta }}+{\dot{\hat{\Theta }}}^{\text{H}}\left(\Theta -\hat{\Theta }\right)\right]\\ & = & -M{\left|z_{1}\right|}^{2}+\frac{1}{2}\left[{\bar{z}}_{1}\left(z_{2}-\phi_{1}\right)+z_{1}\left({\bar{z}}_{2}-{\bar{\phi }}_{1}\right)\right]\\ & & +\frac{1}{2}[{\left(\Theta -\hat{\Theta }\right)}^{\text{H}}\left(\Lambda_{1}-\dot{\hat{\Theta }}\right)+{\left(\Lambda_{1}-\dot{\hat{\Theta }}\right)}^{\text{H}}\left(\Theta -\hat{\Theta }\right)]. \end{array}$$ (12)

**Step 2.** For the subsystem

$$\begin{array}{c} \left\{\begin{array}{c} {\dot{z}}_{1}=z_{2}+\Psi_{1}^{H}\Theta ,\\ {\dot{z}}_{2}=z_{3}+\Psi_{2}^{H}\Theta , \end{array}\right. \end{array}$$ (13)

we consider the Lyapunov function candidate as

$$V_{2}=V_{1}+\frac{1}{2}\bar{\left(z_{2}-\phi_{1}\right)}\left(z_{2}-\phi_{1}\right).$$ (14)

Note that by Lemma 1 and the chain rule

$$\frac{d\phi_{1}}{dt}=\left(\frac{\partial \phi_{1}}{\partial z_{1}}\right)\left(z_{2}+\Psi_{1}^{H}\Theta \right)+\left(\frac{\partial \phi_{1}}{\partial {\bar{z}}_{1}}\right)\left({\bar{z}}_{2}+\Theta^{H}\Psi_{1}\right)+{\left(\frac{\partial \phi_{1}}{\partial \hat{\Theta }}\right)}^{T}\dot{\hat{\Theta }},$$

and

$$\frac{\partial {\bar{\phi }}_{1}}{\partial {\bar{z}}_{1}}=\bar{\frac{\partial \phi_{1}}{\partial z_{1}}},\;\;\frac{\partial {\bar{\phi }}_{1}}{\partial \bar{\hat{\Theta }}}=\bar{\frac{\partial \phi_{1}}{\partial \hat{\Theta }}},$$

the time derivative of *V*_2_ along the subsystem Eq (13) is given by

$$\begin{array}{ll} {\dot{V}}_{2}= & {\dot{V}}_{1}+\frac{1}{2}\{\left({\bar{z}}_{2}-{\bar{\phi }}_{1}\right)[\left(z_{3}-\phi_{2}+\phi_{2}+\Psi_{2}^{\text{H}}\Theta \right)-\left(\frac{\partial \phi_{1}}{\partial z_{1}}\right)\left(z_{2}+\Psi_{1}^{\text{H}}\Theta \right)\\ & -\left(\frac{\partial \phi_{1}}{\partial {\bar{z}}_{1}}\right)\left({\bar{z}}_{2}+\Theta^{\text{H}}\Psi_{1}\right)-{\left(\frac{\partial \phi_{1}}{\partial \hat{\Theta }}\right)}^{\text{T}}\dot{\hat{\Theta }}]+\left(z_{2}-\phi_{1}\right)[\left({\bar{z}}_{3}-{\bar{\phi }}_{2}+{\bar{\phi }}_{2}+\Theta^{\text{H}}\Psi_{2}\right)\\ & -\left(\frac{\partial {\bar{\phi }}_{1}}{\partial z_{1}}\right)\left(z_{2}+\Psi_{1}^{\text{H}}\Theta \right)-\left(\frac{\partial {\bar{\phi }}_{1}}{\partial {\bar{z}}_{1}}\right)\left({\bar{z}}_{2}+\Theta^{\text{H}}\Psi_{1}\right)-{\dot{\hat{\Theta }}}^{\text{H}}\bar{\frac{\partial \phi_{1}}{\partial \hat{\Theta }}}]\}. \end{array}$$ (15)

Defining

$$\{\begin{array}{lll} \phi_{2} & = & -M\left(z_{2}-\phi_{1}\right)-z_{1}-\Psi_{2}^{\text{H}}\hat{\Theta }+\left(\frac{\partial \phi_{1}}{\partial z_{1}}\right)\left(z_{2}+\Psi_{1}^{\text{H}}\hat{\Theta }\right)\\ & & +\left(\frac{\partial \phi_{1}}{\partial {\bar{z}}_{1}}\right)\left({\bar{z}}_{2}+{\hat{\Theta }}^{\text{H}}\Psi_{1}\right)+{\left(\frac{\partial \phi_{1}}{\partial \hat{\Theta }}\right)}^{\text{T}}\Lambda_{2},\\ \Lambda_{2} & = & \Lambda_{1}+\left(z_{2}-\phi_{1}\right)\left(\Psi_{2}-\Psi_{1}\bar{\frac{\partial \phi_{1}}{\partial z_{1}}}\right)+\bar{z_{2}-\phi_{1}}\frac{\partial \phi_{1}}{\partial {\bar{z}}_{1}}\Psi_{1},\\ A_{1} & = & 0,\\ A_{2} & = & \left(z_{2}-\phi_{1}\right)\bar{\left(\frac{\partial \phi_{1}}{\partial \hat{\Theta }}\right)}, \end{array}$$ (16)

and substituting Eqs (16) into (15) yield

$$\begin{array}{ll} {\dot{V}}_{2}= & -M|z_{1}{|}^{2}-M{\left|z_{2}-\phi_{1}\right|}^{2}+\frac{1}{2}\left[\bar{z_{2}-\phi_{1}}\left(z_{3}-\phi_{2}\right)+\left(z_{2}-\phi_{1}\right)\bar{z_{3}-\phi_{2}}\right]\\ & +\frac{1}{2}\left[{\left(\Theta -\hat{\Theta }\right)}^{\text{H}}\left(\Lambda_{2}-\dot{\hat{\Theta }}\right)+{\left(\Lambda_{2}-\dot{\hat{\Theta }}\right)}^{\text{H}}\left(\Theta -\hat{\Theta }\right)\right]\\ & +\frac{1}{2}[A_{2}^{\text{H}}\left(\Lambda_{2}-\dot{\hat{\Theta }}\right)+{\left(\Lambda_{2}-\dot{\hat{\Theta }}\right)}^{\text{H}}A_{2}]. \end{array}$$ (17)

**Step i** (*i* ≥ 3). Assume that in Step *i* − 1, there exist *V*_*i*−1_, *ϕ*_*i*−1_, **Λ**_*i*−1_, **A**_*i*−1_ such that

$$\begin{array}{ll} {\dot{V}}_{i-1}= & -M(|z_{1}{|}^{2}+|z_{2}-\phi_{1}{|}^{2}+\ldots +|z_{i-1}-\phi_{i-2}{|}^{2})\\ & +\frac{1}{2}\left[\bar{\left(z_{i-1}-\phi_{i-2}\right)}\left(z_{i}-\phi_{i-1}\right)+\left(z_{i-1}-\phi_{i-2}\right)\bar{\left(z_{i}-\phi_{i-1}\right)}\right]\\ & +\frac{1}{2}\left[{\left(\Theta -\hat{\Theta }\right)}^{\text{H}}\left(\Lambda_{i-1}-\dot{\hat{\Theta }}\right)+{\left(\Lambda_{i-1}-\dot{\hat{\Theta }}\right)}^{\text{H}}\left(\Theta -\hat{\Theta }\right)\right]\\ & +\frac{1}{2}[A_{i-1}^{\text{H}}\left(\Lambda_{i-1}-\dot{\hat{\Theta }}\right)+{\left(\Lambda_{i-1}-\dot{\hat{\Theta }}\right)}^{\text{H}}A_{i-1}]. \end{array}$$ (18)

In order to analyze the subsystem

$$\begin{array}{c} \left\{\begin{array}{c} {\dot{z}}_{1}=z_{2}+\Psi_{1}^{H}\Theta ,\\ \;\;\;\cdots ,\\ {\dot{z}}_{i}=z_{i+1}+\Psi_{i}^{H}\Theta , \end{array}\right. \end{array}$$ (19)

we introduce the following Lyapunov function candidate as

$$V_{i}=V_{i-1}+\frac{1}{2}\bar{\left(z_{i}-\phi_{i-1}\right)}\left(z_{i}-\phi_{i-1}\right).$$ (20)

Then, the time derivative of *V*_*i*_ along Eq (19) is

$$\begin{array}{ll} V_{i}\;= & {\dot{V}}_{i-1}+\frac{1}{2}\{\bar{z_{i}-\phi_{i-1}}[z_{i+1}-\phi_{i}+\phi_{i}+\Psi_{i}^{\text{H}}\Theta -\sum_{k=1}^{i-1}\left(\frac{\partial \phi_{i-1}}{\partial z_{k}})(z_{k+1}+\Psi_{k}^{\text{H}}\Theta \right)\\ & -\sum_{k=1}^{i-1}(\frac{\partial \phi_{i-1}}{\partial {\bar{z}}_{k}})({\bar{z}}_{k+1}+\Theta^{\text{H}}\Psi_{k})-{\left(\frac{\partial \phi_{i-1}}{\partial \hat{\Theta }}\right)}^{\text{T}}\dot{\hat{\Theta }}-{\dot{\hat{\Theta }}}^{\text{H}}\frac{\partial \phi_{i-1}}{\partial \bar{\hat{\Theta }}}]\\ & +(z_{i}-\phi_{i-1})[{\bar{z}}_{i+1}-{\bar{\phi }}_{i}+{\bar{\phi }}_{i}+\Theta^{\text{H}}\Psi_{i}-\sum_{k=1}^{i-1}\frac{\partial {\bar{\phi }}_{i-1}}{\partial z_{k}}(z_{k+1}+\Psi_{k}^{\text{H}}\Theta )\\ & -\sum_{k=1}^{i-1}\frac{\partial {\bar{\phi }}_{i-1}}{\partial {\bar{z}}_{k}}({\bar{z}}_{k+1}+\Theta^{\text{H}}\Psi_{k})-{\left(\frac{\partial {\bar{\phi }}_{i-1}}{\partial \hat{\Theta }}\right)}^{\text{T}}\dot{\hat{\Theta }}-{\dot{\hat{\Theta }}}^{\text{H}}\bar{\frac{\partial \phi_{i-1}}{\partial \hat{\Theta }}}]\}. \end{array}$$ (21)

Give the definition as follows

$$\{\begin{array}{ll} \phi_{i}\;= & -M(z_{i}-\phi_{i-1})-(z_{i-1}-\phi_{i-2})-\Psi_{i}^{\text{H}}\hat{\Theta }+\sum_{k=1}^{i-1}\left(\frac{\partial \phi_{i-1}}{\partial z_{k}})(z_{k+1}+\Psi_{k}^{\text{H}}\hat{\Theta }\right)\\ & +\sum_{k=1}^{i-1}(\frac{\partial \phi_{i-1}}{\partial {\bar{z}}_{k}})({\bar{z}}_{k+1}+{\hat{\Theta }}^{\text{H}}\Psi_{k})+{\left(\frac{\partial \phi_{i-1}}{\partial \hat{\Theta }}\right)}^{\text{T}}\Lambda_{i}+\Lambda_{i}^{\text{H}}\frac{\partial \phi_{i-1}}{\partial \bar{\hat{\Theta }}}+v_{i},\\ \Lambda_{i}\;= & \Lambda_{i-1}+(z_{i}-\phi_{i-1})(\Psi_{i}-\sum_{k=1}^{i-1}\bar{\frac{\partial \phi_{i-1}}{\partial z_{k}}}\Psi_{k})-\bar{z_{i}-\phi_{i-1}}\sum_{k=1}^{i-1}\frac{\partial \phi_{i-1}}{\partial {\bar{z}}_{k}}\Psi_{k},\\ A_{i}\;= & A_{i-1}+\bar{z_{i}-\phi_{i-1}}\frac{\partial \phi_{i-1}}{\partial \bar{\hat{\Theta }}}+(z_{i}-\phi_{i-1})\bar{\left(\frac{\partial \phi_{i-1}}{\partial \hat{\Theta }}\right)}, \end{array}$$ (22)

where *v*_*i*_ is the auxiliary input to be decided. Noting that

$$A_{i-1}^{H}\left(\Lambda_{i-1}-\Lambda_{i}\right)=A_{i-1}^{H}\left[-\left(z_{i}-\phi_{i-1}\right)\left(\Psi_{i}-\sum_{k=1}^{i-1}\bar{\frac{\partial \phi_{i-1}}{\partial z_{k}}}\Psi_{k}\right)+\bar{z_{i}-\phi_{i-1}}\sum_{k=1}^{i-1}\frac{\partial \phi_{i-1}}{\partial {\bar{z}}_{k}}\Psi_{k}\right],$$ (23)

we take

$$\left\{ \begin{array}{lll} v_{1} & = & v_{2}=0,\\ v_{i} & = & -\left(\Psi_{i}^{\text{H}}-\sum_{k=1}^{i-1}\frac{\partial \phi_{i-1}}{\partial z_{k}}\Psi_{k}^{\text{H}}\right)A_{i-1}+A_{i-1}^{\text{H}}\sum_{k=1}^{i-1}\frac{\partial \phi_{i-1}}{\partial {\bar{z}}_{k}}\Psi_{k}. \end{array}\right.$$ (24)

It follows from Eqs (23) and (24) that

$$\begin{array}{ll} & A_{i-1}^{\text{H}}(\Lambda_{i-1}-\dot{\hat{\Theta }})+{\left(\Lambda_{i-1}-\dot{\hat{\Theta }}\right)}^{\text{H}}A_{i-1}+\bar{\left(z_{i}-\phi_{i-1}\right)}v_{i}+{\bar{v}}_{i}(z_{i}-\phi_{i-1})\\ = & A_{i-1}^{\text{H}}(\Lambda_{i}-\dot{\hat{\Theta }})+{\left(\Lambda_{i}-\dot{\hat{\Theta }}\right)}^{\text{H}}A_{i-1}. \end{array}$$ (25)

Combining Eqs (22)–(25) with Eq (21), we get

$$\begin{array}{lll} {\dot{V}}_{i} & = & -M(|z_{1}{|}^{2}+|z_{2}-\phi_{1}{|}^{2}+\cdots +|z_{i}-\phi_{i-1}{|}^{2})\\ & & +\frac{1}{2}[\bar{\left(z_{i}-\phi_{i-1}\right)}(z_{i+1}-\phi_{i})+(z_{i}-\phi_{i-1})\bar{z_{i+1}-\phi_{i}}]\\ & & +\frac{1}{2}[{\left(\Theta -\hat{\Theta }\right)}^{\text{H}}(\Lambda_{i}-\dot{\hat{\Theta }})+{\left(\Lambda_{i}-\dot{\hat{\Theta }}\right)}^{\text{H}}(\Theta -\hat{\Theta })]\\ & & +\frac{1}{2}[A_{i}^{\text{H}}(\Lambda_{i}-\dot{\hat{\Theta }})+{\left(\Lambda_{i}-\dot{\hat{\Theta }}\right)}^{\text{H}}A_{i}]. \end{array}$$ (26)

**Step n.** Use the same derivation procedure as the above, and assume that we have got *V*_*n*−1_, *ϕ*_*n*−1_, **Λ**_*n*−1_, **A**_*n*−1_, *v*_*n*−1_. Construct the following Lyapunov function candidate as

$$V_{n}=V_{n-1}+\frac{1}{2}\bar{\left(z_{n}-\phi_{n-1}\right)}\left(z_{n}-\phi_{n-1}\right).$$ (27)

Hence, the control input is given by

$$u=-\varphi \left(z,\bar{z},t\right)+\phi_{n},$$ (28)

where

$$\{\begin{array}{lll} \phi_{n} & = & -M(z_{n}-\phi_{n-1})-(z_{n-1}-\phi_{n-2})-\Psi_{n}^{\text{H}}\hat{\Theta }+\sum_{k=1}^{n-1}\left(\frac{\partial \phi_{n-1}}{\partial z_{k}})(z_{k+1}+\Psi_{k}^{\text{H}}\hat{\Theta }\right)\\ & & +\sum_{k=1}^{n-1}(\frac{\partial \phi_{n-1}}{\partial {\bar{z}}_{k}})({\bar{z}}_{k+1}+{\hat{\Theta }}^{\text{H}}\Psi_{k})+{\left(\frac{\partial \phi_{n-1}}{\partial \hat{\Theta }}\right)}^{\text{T}}\Lambda_{n}+\Lambda_{n}^{\text{H}}\frac{\partial \phi_{n-1}}{\partial \bar{\hat{\Theta }}}+v_{n},\\ \Lambda_{n} & = & \Lambda_{n-1}+(z_{n}-\phi_{n-1})(\Psi_{n}-\sum_{k=1}^{n-1}\bar{\frac{\partial \phi_{n-1}}{\partial z_{k}}}\Psi_{k})-\bar{z_{n}-\phi_{n-1}}\sum_{k=1}^{n-1}\frac{\partial \phi_{n-1}}{\partial {\bar{z}}_{k}}\Psi_{k},\\ v_{n} & = & -(\Psi_{n}^{\text{H}}-\sum_{k=1}^{n-1}\frac{\partial \phi_{n-1}}{\partial z_{k}}\Psi_{k}^{\text{H}})A_{n-1}+A_{n-1}^{\text{H}}\sum_{k=1}^{n-1}\frac{\partial \phi_{n-1}}{\partial {\bar{z}}_{k}}\Psi_{k}. \end{array}$$ (29)

Taking

$$A_{n}=A_{n-1}+\bar{\left(z_{n}-\phi_{n-1}\right)}\frac{\partial \phi_{n-1}}{\partial \bar{\hat{\Theta }}}+\left(z_{n}-\phi_{n-1}\right)\bar{\frac{\partial \phi_{n-1}}{\partial \hat{\Theta }}},$$ (30)

and combining Eqs (28) and (29), we have

$$\begin{array}{lll} {\dot{V}}_{n} & = & -M(|z_{1}{|}^{2}+|z_{2}-\phi_{1}{|}^{2}+\cdots +|z_{n}-\phi_{n-1}{|}^{2})\\ & & +\frac{1}{2}\left[{\left(\Theta -\hat{\Theta }\right)}^{\text{H}}\left(\Lambda_{n}-\dot{\hat{\Theta }}\right)+{\left(\Lambda_{n}-\dot{\hat{\Theta }}\right)}^{\text{H}}\left(\Theta -\hat{\Theta }\right)\right]\\ & & +\frac{1}{2}\left[A_{n}^{\text{H}}\left(\Lambda_{n}-\dot{\hat{\Theta }}\right)+{\left(\Lambda_{n}-\dot{\hat{\Theta }}\right)}^{\text{H}}A_{n}\right]. \end{array}$$ (31)

### Stability analysis

**Theorem 1.** Consider the *n* (≥ 3)-dimensional strict-feedback system (7) with initial condition **z**(0), suppose Assumptions 1 and 2 hold. If the adaptive complex scalar controller is designed as

$$u=-\varphi \left(z,\bar{z},t\right)+\phi_{n},$$ (32)

and the complex update law of complex parameter vector **Θ** is chosen as

$$\dot{\hat{\Theta }}=\Lambda_{n},$$ (33)

where *ϕ*_*i*_, **Λ**_*i*_, *A*_*i*_ and the auxiliary input *v*_*i*_ (1 ≤ *i* ≤ *n*) are defined as Eqs (11), (16), (22), (24), (29) and (30), then the controlled system (7) is globally asymptotically stable.

**Proof.** Substitution of $\dot{\hat{\Theta }}$ from Eq (33) into Eq (31) yields

$$\begin{array}{c} {\dot{V}}_{n}=-M(|z_{1}{|}^{2}+...+|z_{n}-\phi_{n-1}{|}^{2})\leq 0. \end{array}$$

By integrating the above inequality from 0 to ∞, we get

$$V_{n}\left(\infty \right)-V_{n}\left(0\right)\leq -M\int_{0}^{\infty }\left(\right|z_{1}{|}^{2}+...+|z_{n}-\phi_{n-1}{|}^{2})dt.$$

It implies that $\int_{0}^{\infty }\left(\right|z_{1}{|}^{2}+...+|z_{n}-\phi_{n-1}{|}^{2})dt$ and *V*_*n*_(∞) are bounded. Since *V*_*n*_(*t*) is continuous, *V*_*n*_(*t*) and |*z*_1_|^2^ + … + |*z*_*n*_ − *ϕ*_*n*−1_|^2^ is bounded. Hence, it can be concluded that |*z*_1_|, |*z*_2_ − *ϕ*_1_|, …, |*z*_*n*_ − *ϕ*_*n*−1_| ∈ *L*_2_ ∩ *L*_∞_.

On the other hand, since $∥\Theta -\hat{\Theta }∥$ is bounded, it follows from Eqs (9) and (10) that $|{\dot{z}}_{1}|$ is also bounded. Hence, *z*_1_ is uniformly continuous. Similarly, *z*_2_ − *ϕ*_1_, …, *z*_*n*_ − *ϕ*_*n*−1_ is uniformly continuous. Therefore, by Lemma 2, *z*_1_ → 0, *z*_2_ − *ϕ*_1_ → 0, …, *z*_*n*_ − *ϕ*_*n*−1_ → 0, as *t* → ∞. Moreover, in view of the continuity of **Ψ**_1_ and **Ψ**_1_(0) = **0** followed from Assumption 1, and noting that the definition of *ϕ*_1_ in Eq (11), we conclude that $\lim_{t\to \infty }\phi_{1}=0$. Thus, *z*_2_ → 0 as *t* → ∞.

Assume that *z*_1_, *z*_2_, …, *z*_*i*_, *ϕ*_1_, *ϕ*_2_, …, *ϕ*_*i*−1_ (*i* ≥ 2) converge to 0 as *t* → ∞. It is clear that **Λ**_*i*_, *A*_*i*_, *v*_*i*_ also converge to **0** or 0 as *t* → ∞, which implies that $\lim_{t\to \infty }\phi_{i}=0$. Noting that *z*_*i*+1_ − *ϕ*_*i*_ → 0 as *t* → ∞, we obtain $\lim_{t\to \infty }z_{i+1}=0$. Repeating this procedure yields $\lim_{t\to \infty }z_{k}=0$, *k* = 1, 2, …, *n*.

Therefore, the controlled system (7) is stabilized. The proof is completed.

**Corollary 1.** Consider 2-dimensional strict-feedback CVCSs

$$\begin{array}{c} \left\{\begin{array}{c} {\dot{z}}_{1}=z_{2}+\Psi_{1}^{H}\left(z_{1},{\bar{z}}_{1}\right)\Theta ,\\ {\dot{z}}_{2}=\varphi \left(z_{1},z_{2},{\bar{z}}_{1},{\bar{z}}_{2},t\right)+\Psi_{2}^{H}\left(z_{1},z_{2},{\bar{z}}_{1},{\bar{z}}_{2},\right)\Theta +u\left(t\right), \end{array}\right. \end{array}$$ (34)

where $z_{1}=z_{1}^{r}+jz_{1}^{i},z_{2}=z_{2}^{r}+jz_{2}^{i}$ are complex-valued state variables. Suppose Assumptions 1 and 2 hold. For given initial condition **z**(0) = (*z*_1_(0), *z*_2_(0))^T^, if the adaptive complex scalar controller is designed as

$$\begin{array}{lll} u\left(t\right) & = & -\varphi -M\left(z_{2}-\phi_{1}\right)-z_{1}-\Psi_{2}^{\text{H}}\hat{\Theta }+\frac{\partial \phi_{1}}{\partial z_{1}}\left(z_{2}+\Psi_{1}^{\text{H}}\hat{\Theta }\right)\\ & & +\left(\frac{\partial \phi_{1}}{\partial {\bar{z}}_{1}}\right)\left({\bar{z}}_{2}+{\hat{\Theta }}^{\text{H}}\Psi_{1}\right)+{\left(\frac{\partial \phi_{1}}{\partial \hat{\Theta }}\right)}^{\text{T}}\Lambda_{2}, \end{array}$$ (35)

and the complex update law of complex parameter vector **Θ** is chosen as

$$\dot{\hat{\Theta }}=\Lambda_{2},$$ (36)

where

$$\begin{array}{c} \left\{\begin{array}{c} \phi_{1}=-Mz_{1}-\Psi_{1}^{H}\hat{\Theta },\\ \Lambda_{2}=z_{1}\Psi_{1}+\left(z_{2}-\phi_{1}\right)\left(\Psi_{2}-\bar{\frac{\partial \phi_{1}}{\partial z_{1}}}\Psi_{1}\right)-\bar{z_{2}-\phi_{1}}\frac{\partial \phi_{1}}{\partial {\bar{z}}_{1}}\Psi_{1}. \end{array}\right. \end{array}$$ (37)

then the controlled system (34) is globally asymptotically stable.

**Proof.** It is similar to the former design procedure and the proof in Theorem 1 and thus is omitted.

**Remark 2.** Theorems 1 and Corollary 1 guarantee the controlled CVCSs Eq (7) to be globally asymptotically stable. Therefore, one can make the controlled system converge to other attractors instead of zero by introducing a appropriate linear transformation of coordinate.

**Remark 3.** Compared with prior work [18–29], we address stabilization of time-dependent strict-feedback CVCSs with uncertain complex parameters and perturbations, and design the adaptive complex scalar controller and complex update laws of uncertain complex parameters. From the technical perspective, as our previous works [30–33], we don’t separate the real and imaginary parts of the complex state variables or complex parameters, and accomplish all the theoretical works in the sense of Wirtinger calculus in complex space. It’s clear that separating imaginary and real parts of complex variables is a frequently used in [18–29], but largely ineffective solution to a stabilization problem in that case the imaginary and real parts cannot be separated.

**Remark 4.** If **Θ** is real-valued parameter vector, Theorems 1 and Corollary 1 are also applied to achieve stabilization of strict-feedback CVCSs with real parameters [28, 29]. However, it’s clear that one cannot stabilize the strict-feedback CVCSs with complex-valued parameters by the method presented in [28, 29].

**Remark 5.** If both the parameters and state variables are taken to be real-valued, Theorems 1 and Corollary 1 are also applied to achieve stabilization of real-variable strict-feedback chaotic systems [12, 14]. However, it’s clear that one cannot stabilize the strict-feedback system with complex-valued parameters and complex-valued state variables by the method presented in [12, 14].

**Remark 6.** It is noted that based on the backstepping method, a recursive design is provided for stabilization problems of a class of CVCSs with **Ψ**_*i*_ ≠ 0, (*i* = 1, 2, …, *n*) in theory. In fact, it is also applicable under the circumstances that some of the terms **Ψ**_*i*_, (*i* = 1, 2, …, *n*) degenerate into zero in the real application.

## Numerical example

**Fig 2:**
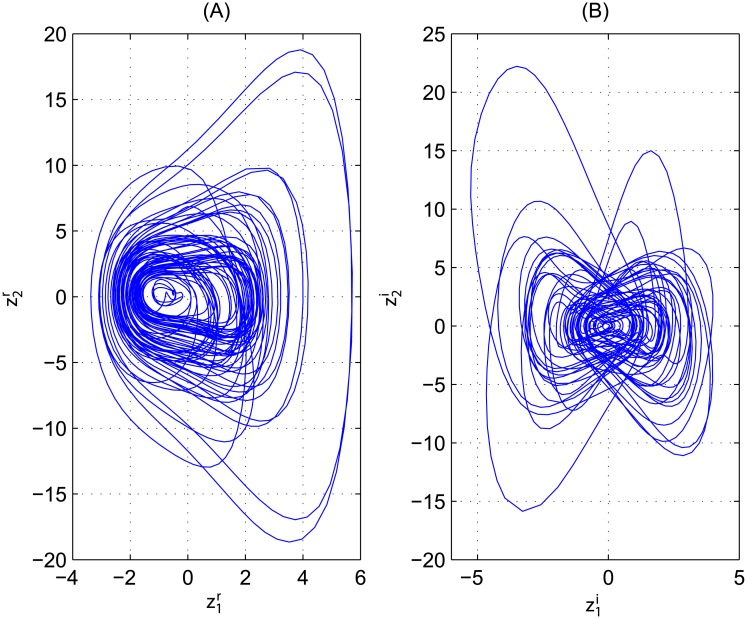
Phase portrait of the chaotic attractor for perturbed Duffing CVCSs Eq (38) when $\psi_{1}=sin\left({\bar{z}}_{1}\right),\;\psi_{2}={\bar{z}}_{1}^{2}+{\bar{z}}_{2}^{2},\;\theta =0.2$ and in the absence of the controller *u*(*t*). (A): On ($z_{1}^{r}$, $z_{2}^{r}$) plane. (B): On ($z_{1}^{i}$, $z_{2}^{i}$) plane.

In this section, we take the Duffing CVCSs as an example to verify and demonstrate the effectiveness of the proposed control scheme. The simulation results are carried out using the MATLAB software. The fourth order Runge-Kutta integration algorithm was performed to solve the differential equations. The 2-dimensional Duffing CVCSs Eq (1) is perturbed by uncertainty terms $\psi_{i}^{H}\theta ,\left(i=1,2\right)$, which is described as follows

$$\begin{array}{c} \left\{\begin{array}{c} {\dot{z}}_{1}=z_{2}+\psi_{1}^{H}\left(z_{1},{\bar{z}}_{1}\right)\theta ,\\ {\dot{z}}_{2}=f\left(z_{1},z_{2},{\bar{z}}_{1},{\bar{z}}_{2},t\right)+\psi_{2}^{H}\left(z_{1},z_{2},{\bar{z}}_{1},{\bar{z}}_{2}\right)\theta +u\left(t\right), \end{array}\right. \end{array}$$ (38)

where $z_{1}=z_{1}^{r}+jz_{1}^{i},z_{2}=z_{2}^{r}+jz_{2}^{i}$ are complex-valued state variables, $f=z_{1}-\alpha z_{2}-\beta z_{1}^{2}{\bar{z}}_{1}+\gamma^{\prime }cos\left(\omega t\right)$, $\gamma^{\prime }=\sqrt{2}\gamma \exp\left(\frac{j\pi }{4}\right)$, *α*, *β*, *γ* and *ω* are positive parameters, *θ* is uncertain complex parameter, and *u*(*t*) = *u*^*r*^(*t*) + *ju*^*i*^(*t*) is the control input. The control-free perturbed complex system (38) is also chaotic in Fig 2 when $\psi_{1}=sin\left({\bar{z}}_{1}\right),\;\psi_{2}={\bar{z}}_{1}^{2}+{\bar{z}}_{2}^{2},\;\theta =0.2$, and at the same values of the parameters and initial conditions as in Fig 1.

According to Eqs (35)–(37) in Corollary 1, the adaptive complex scalar controller is constructed as follows.

$$\begin{array}{lll} u\left(t\right) & = & -f-M\left(z_{2}-\phi_{1}\right)-z_{1}-\left(z_{1}^{2}+z_{2}^{2}\right)\hat{\theta }\\ & & -\left(M+\hat{\theta }cosz_{1}\right)\left(z_{2}+\hat{\theta }sinz_{1}\right)-\lambda_{2}sinz_{1}, \end{array}$$ (39)

and the complex update law of complex parameter *θ* is given by

$$\dot{\hat{\theta }}=\lambda_{2},$$ (40)

where

$$\begin{array}{c} \left\{\begin{array}{c} \phi_{1}=-Mz_{1}-\hat{\theta }sinz_{1},\\ \lambda_{2}=z_{1}sin{\bar{z}}_{1}+\left(z_{2}-\phi_{1}\right)\left[{\bar{z}}_{1}^{2}+{\bar{z}}_{2}^{2}+\left(M+\bar{\hat{\theta }}cos{\bar{z}}_{1}\right)sin{\bar{z}}_{1}\right]. \end{array}\right. \end{array}$$ (41)

**Fig 3:**
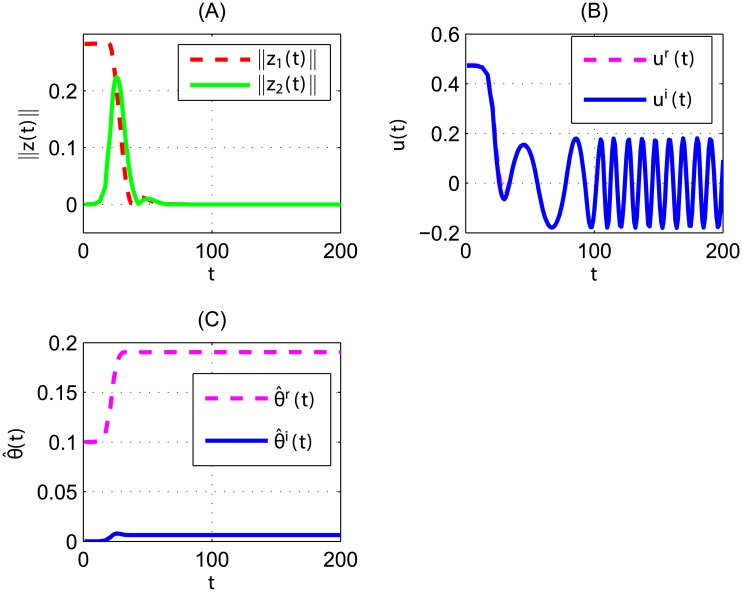
Time response for controlled Duffing CVCSs Eq (38) with the adaptive complex scalar controller Eq (39) and complex update law Eqs (40) and (41), and at the same parameter values and initial conditions as in Fig 1. (A): Time response of the complex state variables $z_{1}=z_{1}^{r}+jz_{1}^{i},z_{2}=z_{2}^{r}+jz_{2}^{i}$. (B): Time response of the complex scalar controller *u*(*t*) = *u*^*r*^(*t*) + *ju*^*i*^(*t*). (C): Time response of uncertain complex parameter estimation $\hat{\theta }={\hat{\theta }}^{r}+j{\hat{\theta }}^{i}$.

In the numerical simulations, $M=1,\;\hat{\theta }\left(0\right)=0.1$, and the same values of the other parameters and initial conditions are chosen as in Fig 1. The state variables *z*_1_ and *z*_2_ of system (38) converge asymptotically to zero as demonstrated in Fig 3(A), where the dotted red line shows mode of the variable *z*_1_ and the solid green line presents mode of the variable *z*_2_. The time response of complex scalar controller and uncertain complex parameter estimation $\hat{\theta }$ are shown in Fig 3(B) and 3(C), where the dotted pink line shows the real part of the control input (parameter) and the solid blue line the imaginary part of the control input (parameter).

Therefore, with the adaptive complex scalar controller Eq (39) and complex update law Eqs (40) and (41), system (38) is stabilized. And note that the control input is periodical after some time (the imaginary part of the controller is very the same as the real part when t is sufficiently large) as in Fig 3(B). In fact, $\lim_{∥z∥\to 0}u=\gamma^{\prime }cost=\left(0.18+0.18j\right)cost$.

**Remark 7.** The persistent exiting conditions (PE conditions) in real space could not be easily extended to complex space because there exist neither sign function nor comparison of complex numbers in the complex field. It is difficult or even impossible to solve the precise parameter estimate problem in complex space by the existing approaches. Therefore, it is a challenging but crucial issue, and we will continue the topic in the near future.

## Discussion and conclusions

In this paper, we have developed a new unified framework for the stabilization of a class of *n*-dimensional time-dependent strict-feedback CVCSs with uncertain complex parameters and perturbations. In detail, appropriate Lyapunov functions dependent on complex-valued vectors and unknown complex parameters have been constructed, and their Lie derivatives are provided in the sense of Wirtinger calculus. And on this basis, an efficient back-stepping design has been proposed for controlling this type of CVCSs. It should be noted that it needs only one complex scalar controller to realize stabilization no matter how many dimensions CVCSs contain and the conditions for the existence of controller are very easy to check. Especially, this method combine the identification of unknown complex parameters with back-stepping design to control time-dependent strict-feedback CVCSs. The proposed systematic procedure sheds some light on the potential real world applications, such as the electronic and mechanic devices, biology and medicine, and so on.
